# The Sphingosine-1-Phosphate Lyase (LegS2) Contributes to the Restriction of *Legionella pneumophila* in Murine Macrophages

**DOI:** 10.1371/journal.pone.0146410

**Published:** 2016-01-07

**Authors:** Arwa Abu Khweek, Apurva Kanneganti, Denis C. Guttridge D, Amal O. Amer

**Affiliations:** 1 Birzeit University, Department of Biology and Biochemistry, West Bank, Palestine; 2 The Ohio State University, Department of Microbial Infection and Immunity, College of Medicine, Center for Microbial Interface Biology, Columbus, Ohio, United States of America; 3 Human Cancer Genetics Program, Ohio State University, Columbus, Ohio, United States of America; UC Irvine Medical Center, UNITED STATES

## Abstract

*L*. *pneumophila* is the causative agent of Legionnaires’ disease, a human illness characterized by severe pneumonia. In contrast to those derived from humans, macrophages derived from most mouse strains restrict *L*. *pneumophila* replication. The restriction of *L*. *pneumophila* replication has been shown to require bacterial flagellin, a component of the type IV secretion system as well as the cytosolic NOD-like receptor (NLR) Nlrc4/ Ipaf. These events lead to caspase-1 activation which, in turn, activates caspase-7. Following caspase-7 activation, the phagosome-containing *L*. *pneumophila* fuses with the lysosome, resulting in the restriction of *L*. *pneumophila* growth. The LegS2 effector is injected by the type IV secretion system and functions as a sphingosine 1-phosphate lyase. It is homologous to the eukaryotic sphingosine lyase (SPL), an enzyme required in the terminal steps of sphingolipid metabolism. Herein, we show that mice Bone Marrow-Derived Macrophages (BMDMs) and human Monocyte-Derived Macrophages (hMDMs) are more permissive to *L*. *pneumophila legS2* mutants than wild-type (WT) strains. This permissiveness to *L*. *pneumophila legS2* is neither attributed to abolished caspase-1, caspase-7 or caspase-3 activation, nor due to the impairment of phagosome-lysosome fusion. Instead, an infection with the *legS2* mutant resulted in the reduction of some inflammatory cytokines and their corresponding mRNA; this effect is mediated by the inhibition of the nuclear transcription factor kappa-B (NF-κB). Moreover, BMDMs infected with *L*. *pneumophila legS2* mutant showed elongated mitochondria that resembles mitochondrial fusion. Therefore, the absence of LegS2 effector is associated with reduced NF-κB activation and atypical morphology of mitochondria.

## Introduction

The facultative intracellular pathogen *L*. *pneumophila* multiplies within human alveolar macrophages and modulates host cell signaling. Following internalization by macrophages or amoeba, *L*. *pneumophila* form a unique compartment called the *Legionella* containing vacuole (LCV) that evades fusion with lysosomes [1–7]. The LCV provides the bacteria with a protected environment in which *L*. *pneumophila* can secure replication. The Dot/Icm type IV secretion system, which translocates effector proteins into the host cell cytoplasm and manipulates host cell signaling, coordinates the formation of the LCV [7–9]. Several injected effectors have been previously identified as substrates of the Dot/Icm secretion system [10–14]. Some effectors are involved in the recruitment of the endoplasmic reticulum vesicles to the LCV and thus disrupting host trafficking [15–20]. Others are modulators of the NF-κB pathway [20,21]. Some proteins are homologous to eukaryotic proteins and have domains with enzymatic activity required in various post-translational modifications, including phosphorylation, glycosylation, methylation, prenylation, AMPylation and ubiquitination, of host cell proteins [14,20,22–26].

The subcellular compartments in eukaryotic cells are designated by their lipids and proteins content. Trafficking is tightly controlled to guarantee the correct delivery of cargo to the correct compartment [27]. For example, phosphatidylinositol 4- phosphate PtdIns (4)P and phosphatidylinositol 3- phosphate PtdIns (3)P have been shown to regulate phagolysosome biogenesis [28]. Some *L*. *pneumophila* secreted effectors anchor to the cytoplasmic face of the LCV membrane by binding to phosphoinositide (PI) lipids [29]. This process is achieved through the modulation of the vacuole membrane PI pattern such as the accumulation of PtdIns (4)P, which is catalyzed by *L*. *pneumophila* effector proteins that directly manipulate PIs or indirectly control them through effectors that recruit host PI-metabolizing enzymes [27,29]. Moreover, *L*. *pneumophila* largely controls the localization of secreted bacterial effectors and the recruitment of host factors by modulating the PI patterns at the LCV.

The *legS2* (lpg2176) was identified in a bioinformatics screen of the *L*. *pneumophila* Philadelphia-1 genome and encodes for a protein that is highly homologous to the eukaryotic sphingosine 1-phosphate lyase [30,31]. LegS2 exhibits 36% identity and 52% similarity to *Tetrahymena thermophila* SPL and functions as a sphingosine 1-phosphate lyase [31]. The *L*. *pneumophila* SPL harbors a C-terminal domain that is required for translocation to eukaryotic cells via the Icm/Dot system [31]. This domain is absent in the eukaryotic homologues.

SPL is required for the degradation of Sphingosine-1-Phosphate S1P to phosphoethanolamine and hexadecanal in eukaryotic cells [31]; Sphingosine-1-Phosphate S1P is a sphingolipid metabolite that regulates cell migration, angiogenesis, and development [32]. The intracellular pool of S1P is regulated by three highly conserved enzymes: sphingosine kinase (SPHK) that catalyzes the phosphorylation of sphingosine producing S1P, S1P phosphatase (S1PP) that reverses the former reaction, and S1P lyase (SPL) that catalyzes the irreversible cleavage of S1P to ethanolamine phosphate and a long chain aldehyde [32].

In this study, we show that *L*. *pneumophila* lacking the sphingosine-1-phosphate lyase, *legS2* replicated in higher numbers compared to WT strain in WT BMDMs. The increase in bacterial multiplication is not attributed to the compromised activation of caspase-1, caspase-7, or caspase-3 or phagosome lysosome fusion. Instead, the disruption of *L*. *pneumophila* SPL is associated with a reduction in the level of inflammatory cytokines. This reduction results from the inhibition of the NF-κB pathway. Moreover, infection with the *legS2* mutant results in an elongation of the mitochondria that is a characteristic of mitochondrial fusion.

## Materials and Methods

### Ethics statements

This study was carried out in strict accordance with the recommendations in the Guide for the Care and Use of Laboratory Animals of the National Institute of health and The Ohio State University. The Institutional Animal Care and Use Committee (IACUC) has approved our Animal Use Protocol (AUP), 2010A00000066-R1 and approved this study. Approved by Donna McCarthy Beckett, Ph.D., R.N., F.A.A.N. Human cells were obtained from anonymous samples from the Red Cross, **http://www.redcrossblood.org/centralohio**. Blood from the red cross does not have any information or link to the donor and therefore does not require IRB or consent. Primary macrophages derived from mice bone marrow were used.

### Bacterial strains

*L*. *pneumophila* JR32 [33] and the *legS2* mutant [30] were kindly provided by Dr. Amal Amer, at The Ohio State University. *L*. *pneumophila* strains were grown on buffered charcoal yeast extract solid media (BCYE) plates at 37°C, or in BYE broth containing L-cysteine, thymidine and ferric nitrate supplements at 37°C with shaking [2,34].

### Mice, cell culture and infections

Wild-type mice were purchased from Jackson laboratory. Mice were housed in AALAC-accredited facilities and kept in ventilated cages with automatic water valves (lixit). Mice were fed Harlan 7912 as needed and kept in 12hr light/dark cycles 6am-6pm light, 6pm-6am—dark for their circadian rhythms. Plastic houses/huts were provided for enrichment if one mouse per cage or mice needed to feel more secure. Mice were euthanized by CO_2_ then cervical dislocation. Anesthesias for *in vivo* experiments were done by exposing the mice to isoflurane gas for 30 seconds until they are asleep. Mice were monitored daily for signs of pain or distress. All of the animals received humane care according to the criteria outlined in the Guide for the Care and Use of Laboratory Animals published by National Institutes of Health and The Ohio State University. C57BL/6 BMDMs were prepared from the femurs of five to eight-week-old mice as previously described [2,35] and grown in IMDM (Iscove’s modified Dulbecco’s medium supplemented with the L929 fibroblast cell line (ATCC)-cultured supernatant and 10% heat inactivated fetal bovine serum (HIFBS) (Gibco). Infection was done in IMDM supplemented with 10% HIFBS. The isolation and preparation of the hMDMs from peripheral blood (obtained from the Red Cross) was carried out as previously described [34,36,37].

### *In vitro* and *in vivo* infection

The *in vitro* BMDM infections were carried with a multiplicity of infection (MOI) of 0.2–0.5 unless otherwise specified. For *in vivo* experiments, C57BL/6 mice were infected with 1x10^6^ bacteria intratracheally. The lungs were harvested 4 or 48 h post-infection, homogenized, and plated on BCYE with streptomycin. Colony forming units (CFUs) were then quantified.

### Western blot

Cell extracts were prepared and immunoblotted with antibodies that recognize caspase-1, caspase-7 and caspase-3 (Cell Signaling), followed by the appropriate secondary antibody. Equal concentration of protein was loaded on a 12% SDS polyacrylamide gel.

### Macrophage cytotoxicity assay

*In vitro* quantification of cytoplasmic histone-associated-DNA-fragments (apoptosis) was performed using the Cell Death Detection ELISA and the photometric enzyme immunoassay (Roche Applied Science) according to the manufacturer’s specifications. The fold change of macrophage necrosis was determined by measuring the release of host cell cytoplasmic lactate dehydrogenase (LDH) using the cytotoxicity detection kit (Roche Applied Science) according to the manufacturer’s specifications. BMDMs (0.5 x 10^6^) were plated in 12-well plates and infected with either JR32 or the *legS2* mutant at an MOI of 0.5, 1, or 5. The supernatants were then collected 24 h post-infection and assayed.

### NF-κB DNA binding activity assay

Nuclear extracts of WT BMDMs untreated or treated with the JR32 or *legS2* mutant were prepared as described [2]. EMSA was used to measure NF-κB DNA binding activity as noted [38].

### Lyso tracker red colocalization

Wild-type mouse macrophages were plated at a density of 0.5 x 10^6^ cells per well in a 24 well plate containing sterile coverslips for 48 h. The lyso tracker red was added to the monolayer for 30 min at 37°C. The monolayer was then washed with the infection medium 3 times and kept for 30 min at 37°C in infection media alone. Next, it was infected with JR32, *dotA*, or *legS2* at an MOI of 0.2 for 1 h. Then, the monolayer was washed with the infection medium, fixed with paraformaldehyde, and finally stained with the anti-*L*. *pneumophila* antibody, followed by the secondary antibody Alexa flour 488 goat anti-mouse. The coverslips were mounted and viewed by the Olympus FV1000 spectral Confocal Laser Scanning Microscopy (CLSM).

### Enzyme-linked immuno sorbent assay (ELISA)

Macrophages were infected with *L*. *pneumophila* JR32 or the *legS2* mutant for 24 h. Then, culture supernatants were collected, centrifuged and stored at -20°C until assayed for cytokine content. The amount of IL-1β, IL-6, IL10 and TNFα in the supernatants was determined by specific sandwich ELISA following the manufacturer’s protocol (R&D system Inc.,

### Quantitative PCR

Total RNA was extracted from mouse macrophages using Trizol (Invitrogen Life Technologies). Then, 1–2 μg of the RNA were converted to cDNA by ThermoScript RNase H- Reverse Transcriptase (Invitrogen, Life Technologies). 20–60 ng of the converted cDNA was used for quantitative PCR with SYBR Green I PCR Master Mix in the Step One Plus Real Time PCR System (Applied Biosystems). The target gene Ct values were normalized to the Ct values of two housekeeping genes (human GAPDH and CAP-1, accordingly to the cell origin) and expressed as relative copy number (RCN) (34).

### Transmission Electron Microscopy

BMDMs were plated at a density of 4x10^6^ per well on coverslips in Permanox chamber slides, with 2 chambers and 8 units per tray (lab tech) for 48 h; they were then infected with an MOI of 1 for 4 h or MOI of 0.5 for 24 h. The coverslips were fixed with 2.5% glutaraldehyde in 0.1M phosphate buffer and 0.1M sucrose at a pH of 7.4. The cells were post-fixed with 1% osmium tetroxide in the phosphate buffer; then, they were en bloc stained with 2% uranyl acetate in 10% ethanol, dehydrated in a graded series of ethanol, and embedded in Eponate 12 epoxy resin (Ted Pella Inc., 18012). Ultrathin sections were cut on a Leica EM UC6 ultramicrotome (Leica microsystems, EM FC7), collected on copper grids and stained. Images were acquired with an FEI Technai G_2_ Spirit transmission electron microscope (FEI), a Macrofire (Optronics) digital camera, and AMT image capture software.

### Statistical analysis

The comparisons of groups for statistical difference were done using a Student’s two-tailed t-test. *P* values less than 0.05 were considered significant.

## Results

### *L*. *pneumophila legS2* strain replicates in WT BMDMs and hMDMs

**Fig 1:**
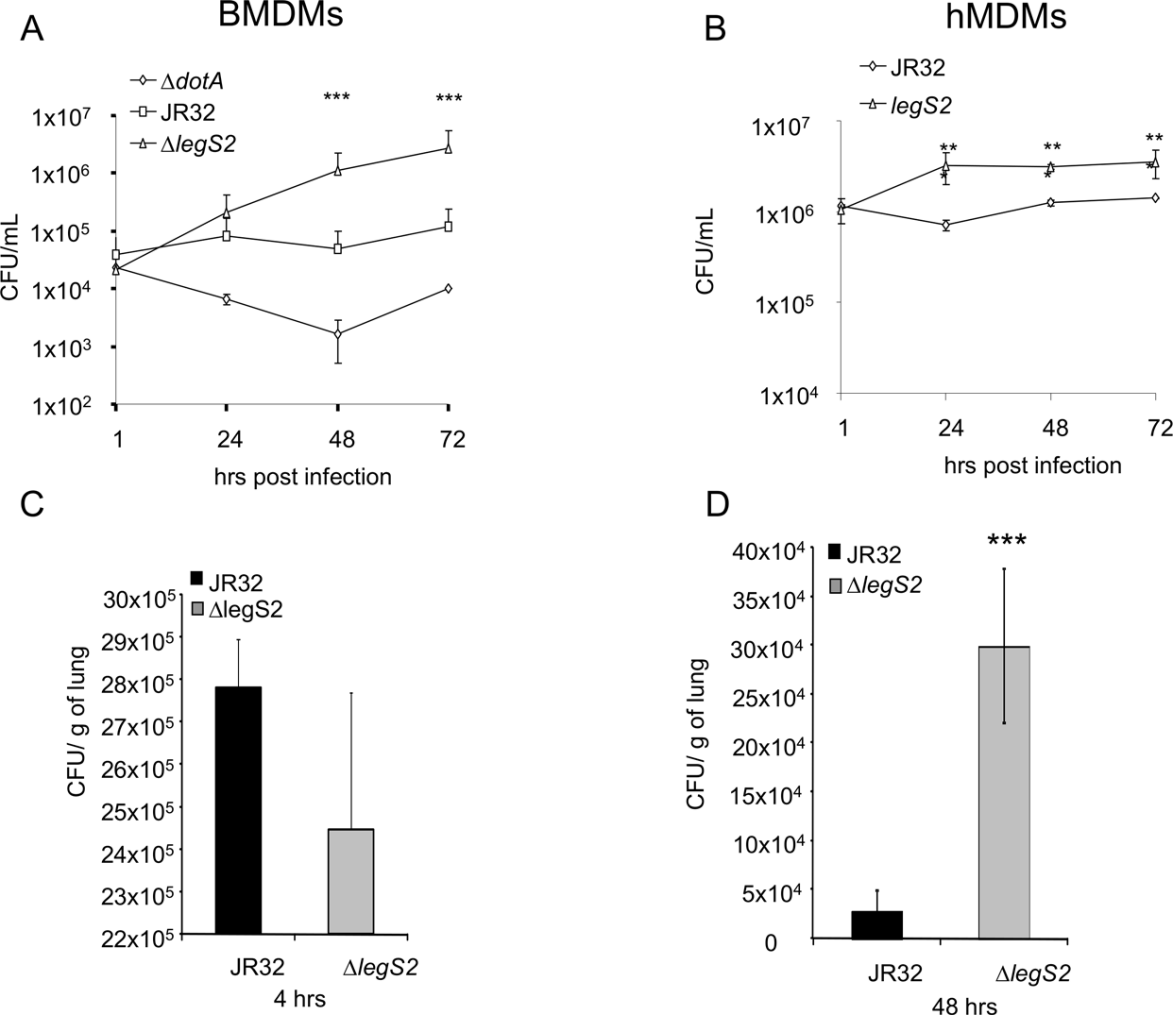
*L*. *pneumophila legS2* mutant replicates in human MDMs and wild-type mice and their derived macrophages. A) BMDMs were infected with wild-type *L*. *pneumophila*, JR32, or *ΔlegS2* for 1, 24, 48 and 72h. The colony forming units (CFUs) were scored at the indicated time points. B) hMDMs were infected with wild-type *L*. *pneumophila*, JR32, or *ΔlegS2* for 1, 24, 48 and 72h. Data are shown as mean + SD of n = 3. Asterisks indicate significant differences, and a two tailed t-test was used to calculate the *P* value (*P<0.05, **P<0.01, ***P<0.001). (C and D) Four female C57BL/6 mice per group received 1x10^6^ of JR32 or *legs2* bacteria intratracheally. Lungs were homogenized and plated for CFUs, counting at (C) 4 or (D) 48 h post infection. (C and D) Data are shown as mean + SD of four mice. Asterisks indicate significant differences (***P<0.001); a two tailed t-test was used to calculate the *P*-value.

To delineate the role of LegS2 in *L*. *pneumophila* infection, we tested the replication of the JR32 and *legS2* mutant in WT BMDMs. The BMDMs restricted the growth of the JR32 strain, while the *legS2* mutant showed a 10- fold increase in the colony forming units (CFUs) over time (48–72 h) compared to the JR32 strain (Fig 1A). Since humans are permissive to *L*. *pneumophila* replication (39), we also examined the intracellular growth of *legS2* mutant in hMDMs (Fig 1B). Both JR32 and *legS2* mutant replicated in hMDMs; however, the *legS2* mutant replicated more than the JR32 strain.

Legionnaires’ disease stems from the successful replication of *L*. *pneumophila* in human alveolar macrophages [39,40]. Therefore, we tested *legS2* mutant replication *in vivo* by examining the bacterial CFUs within the lungs of infected mice. C57BL/6 mice were infected with either 1x10^6^ JR32 or *legS2* mutant intratracheally. The initial bacterial load was not significantly different 4 h post-infection (Fig 1C). However, 48 h post-infection, the *legS2* mutant-infected mice harbored 4 fold more CFUs compared to the JR32 strain (Fig 1D). Our data showed that the *legS2* mutant replicates in WT mice and their derived macrophages.

### The growth of *legs2* mutant is independent of caspase-1 or caspase-7 activation

**Fig 2:**
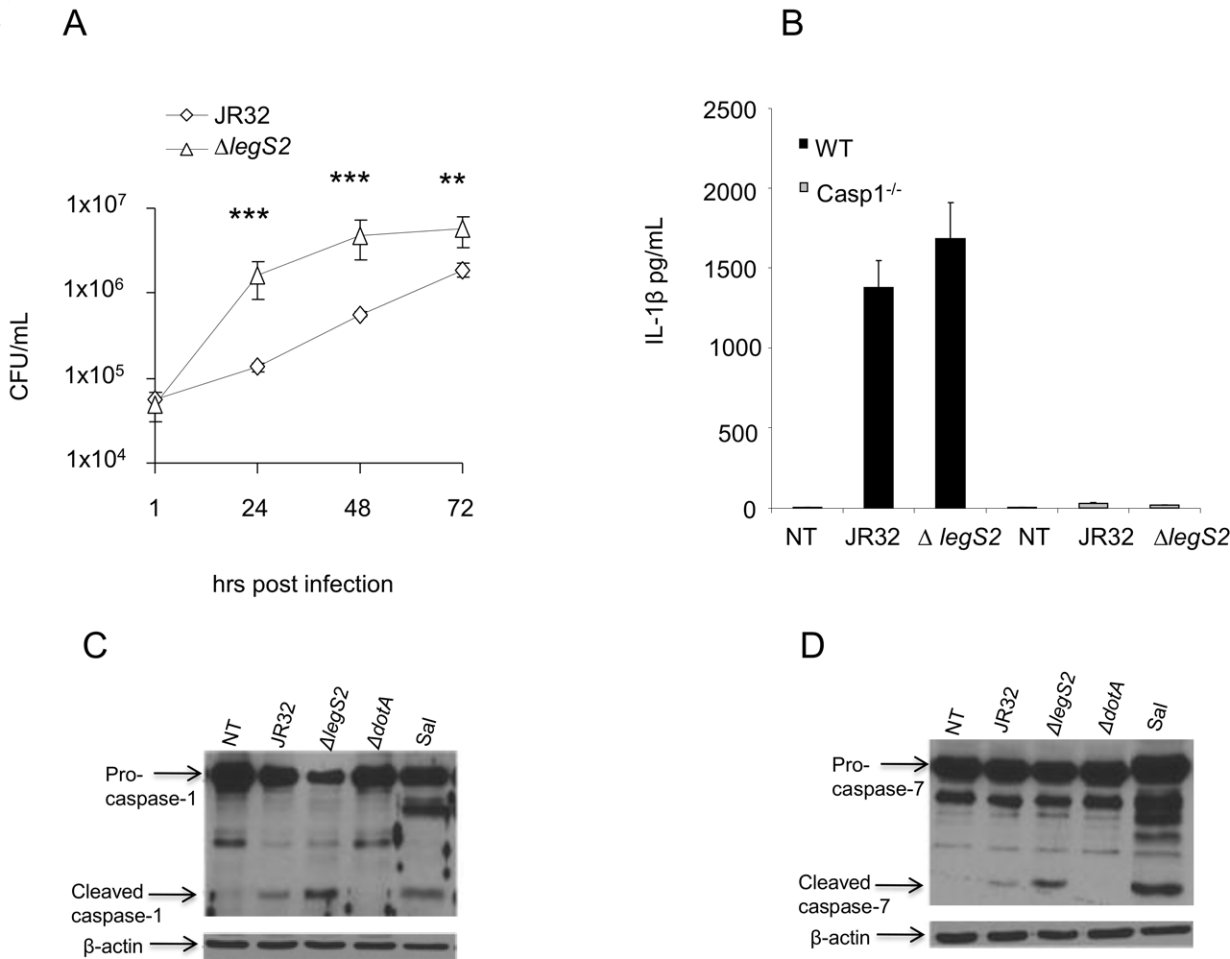
The permissiveness of mice BMDMs to *legS2* mutant bacteria is independent of caspase-1 or caspase-7 activation. (A) Caspase-1 KO (casp-1^-/-^) macrophages were infected with *L*. *pneumophila*, JR32, or *legS2* with an MOI of 0.5 for 1, 24, 48 and 72 h; then, CFUs were measured at the indicated time points. (B) Levels of IL-1β were detected in supernatants of WT or casp-1^-/-^ BMDMs were infected with JR32 or the *legS2* mutant after 24 hr, while WT BMDMs were either not treated (NT) or infected with *L*. *pneumophila*, JR32, or *legS2* mutant bacteria for 2 h. Salmonella infection (Sal) was used as a positive control for caspase-1 or caspase-7 activation. (C) Casp-1 or (D) casp-7 antibodies were used to detect casp-1 and casp-7 activation, respectively, in cell extracts.

Caspase-1 activates caspase-7, resulting in the restriction of *L*. *pneumophila* growth by mediating the fusion of the LCV to the lysosome [41–43]. Casp-1^-/-^ BMDMs are permissive to *L*. *pneumophila* replication [2]. Therefore, impaired caspase-1 activation could result in the increased replication of the *legS2* mutant. Therefore, we examined the replication of *legS2* mutant bacteria in Casp-1^-/-^ BMDMs. The *legS2* mutant showed higher replication than JR32 24, 48, and 72 h post-infection in casp-1^-/-^BMDMs (Fig 2A). Since the cleavage and maturation of IL-1β is mediated by caspase-1 activation, we examined the level of IL-1β 24 h post-infection with the JR32 or the *legS2* mutant by ELISA. No significant differences were seen in the level of IL-1β 24 h post-infection with either the JR32 or the *legS2* mutant (Fig 2B). Moreover, we tested the possibility of differential activation of caspase-1 by the WT JR32 or *legS2* mutant. Indeed, both JR32 and *legS2* infected BMDMs showed capsase-1 cleavage as detected by the mature subunits of casp-1 (Fig 2C). Taken together, the replication of the *legS2* mutant in WT macrophages is independent of caspase-1 activation.

The growth restriction of *L*. *pneumophila* is also attributed to caspase-7 mediated bacterial delivery to the lysosome and the early death of murine macrophages [2,44]. Hence, impaired caspase-7 activation could result in increased replication of the *legS2* mutant. To that end, we assessed the activation of caspase-7 by Western blot. Both the JR32 and the *legS2* mutant cleaved caspase-7 in WT macrophages (Fig 2D). Thus, the replication of the *legS2* mutant is independent of caspase-1 or caspase-7 activation.

### The growth of *legs2* mutant is independent of host cell death

**Fig 3:**
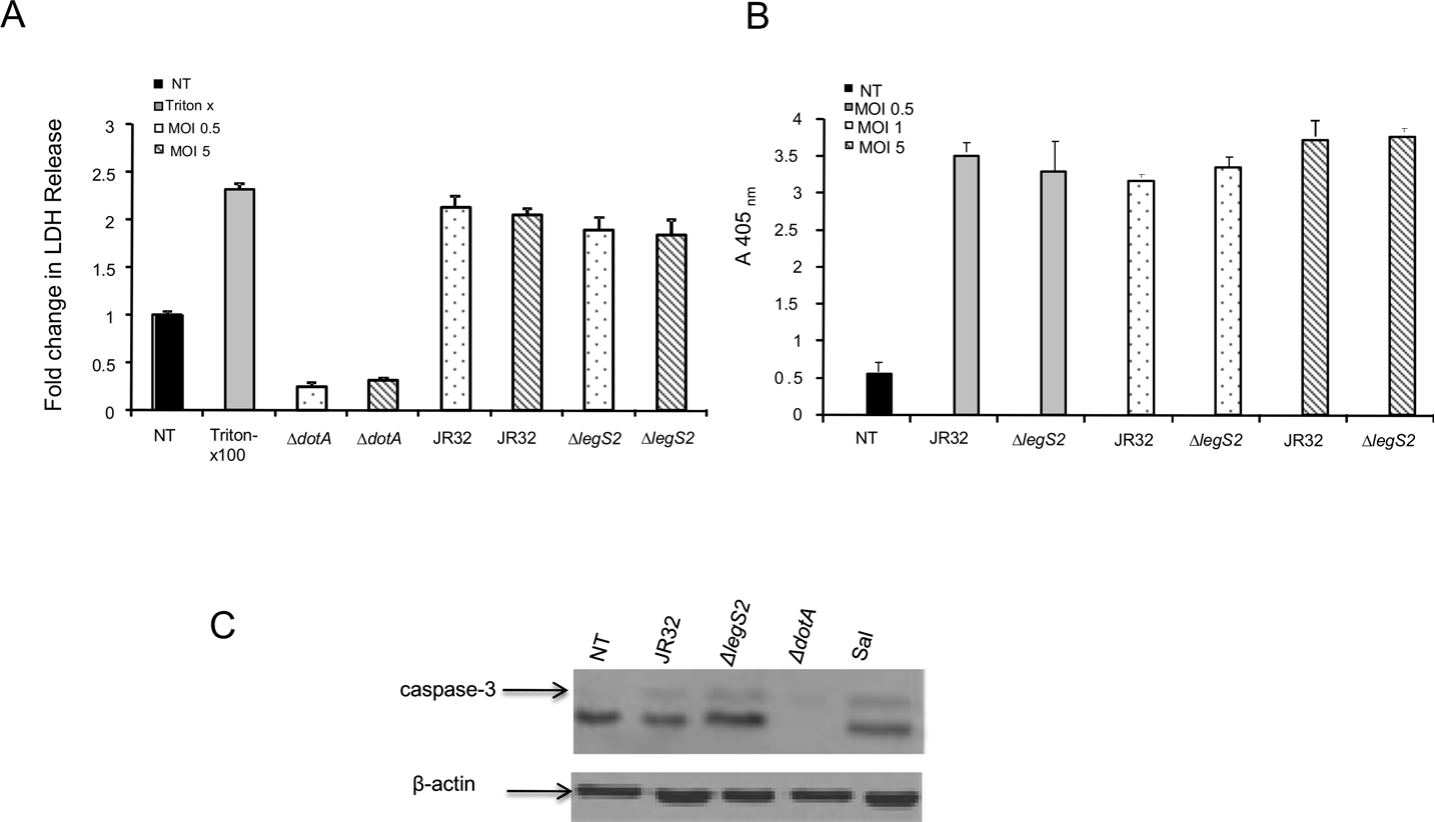
Replication of *legS2* mutant is independent of host cell death. (A) Wild-type BMDMs were not treated (NT) or infected with the type IV secretion mutant *dotA*, wild-type *L*. *pneumophila*, JR32, or *legS2* mutant for 24h at MOIs of 0.5 and 5. Then, the fold change in LDH release was measured from the overall population of macrophages. The data represents the mean + SD of n = 3. (B) Wild-type C57BL/6 (B6) were not treated (NT) or infected with wild-type *L*. *pneumophila*, JR32, *legS2*, or *dotA* mutant for 8 h. Salmonella infection (Sal) was used as a positive control for caspase-3 activation. A Western blot with caspase-3 antibody was used to detect casp-3 activation. β-actin was used as a loading control.

Interference with the host’s survival is a key determinant in the *L*. *pneumophila* replication cycle. Early cell death aborts *L*. *pneumophila* replication and terminates the infection [45]. It is possible that the *legS2* mutant modulates the macrophage’s life span to sustain its replication. To dissect the role of LegS2 in mediating host cell death, we quantified the level of LDH released in the culture supernatants of macrophages infected with either JR32 or the *legS2* mutant. Comparable LDH release was observed in the culture supernatants of both the JR32 and the *legS2* mutant-infected macrophages at the two different MOIs (Fig 3A). Moreover, we measured apoptosis in the overall population of macrophages by determining the cytoplasmic apoptosis histone-associated-DNA fragments at MOIs of 0.5, 1 and 5. We found that infection by JR32 and the *legS2* mutant leads to comparable cell death at both low and high MOIs (Fig 3B). Overall, our data demonstrates that the replication of the *legS2* mutant in macrophages is independent of cell death. Further, the activation of caspase-3 is comparable in JR32 and *legS2* infected macrophages (Fig 3C). Consequently, the replication of the *legS2* mutant is independent of host cell death.

### The replication of the *legS2* mutant in WT BMDM is not attributed to impaired phagosome-lysosome maturation

**Fig 4:**
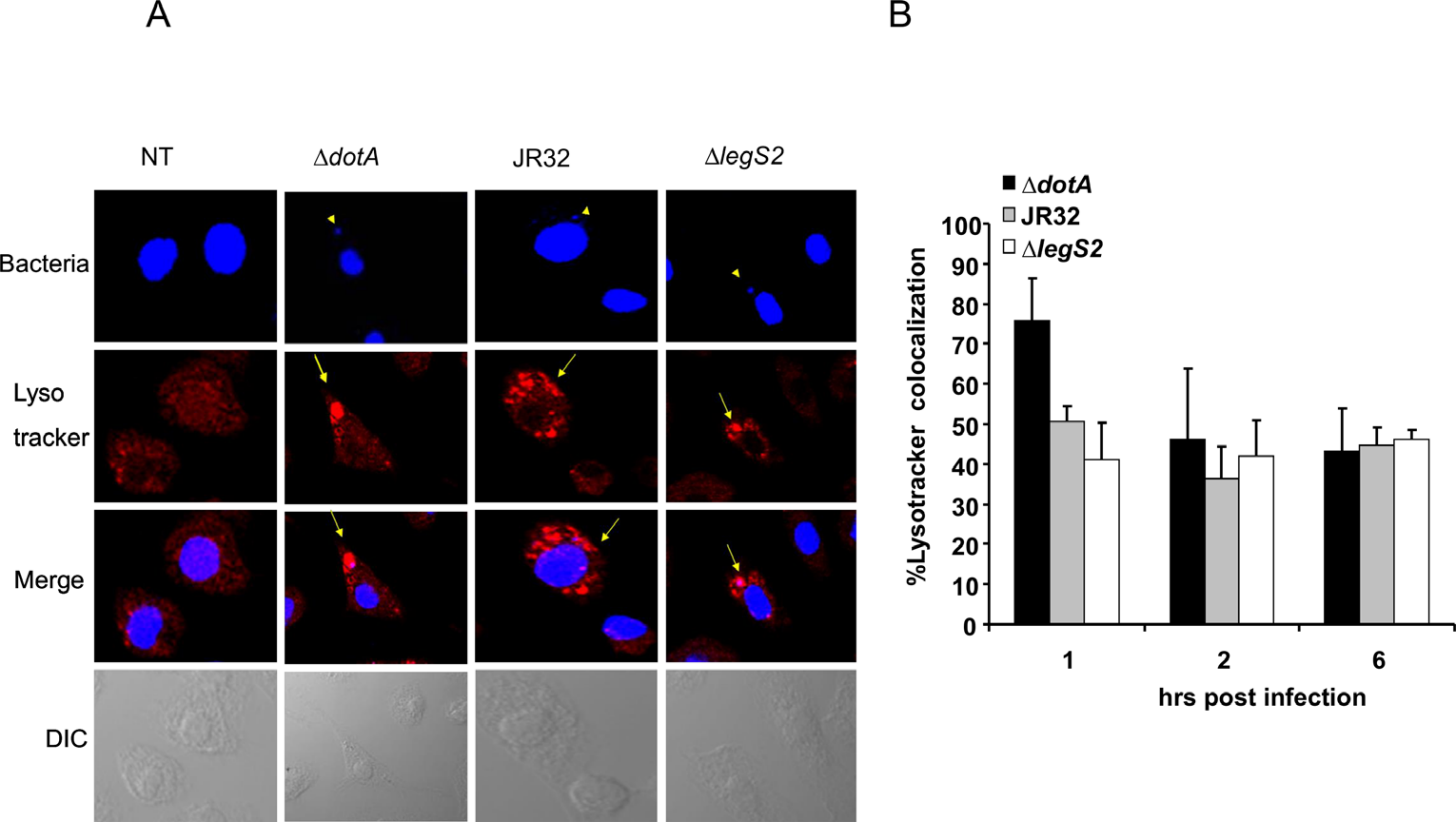
Replication of *L*. *pneumophila legS2* mutant is not due to defective phagosome-lysosome fusion. (A) Images of wild-type macrophages not infected (NT) or infected with the type IV secretion mutant *dotA*, wild-type *L*. *pneumophila*, JR32, and the *legS2* mutant. The first panel presents staining with DAPI, with arrow heads pointing to the bacteria. The second panel shows staining with the lyso tracker. The third panel depicts merged images, with bacteria colocalized with the lysosomal marker. B) The percent of bacteria colocalized with the lyso tracker was scored in 100 infected cells from 3 independent coverslips. The data represents the mean + SD of n = 3.

The restriction of *L*. *pneumophila* in WT BMDMs is dependent upon the delivery of the LCV to the lysosome, which ultimately results in bacterial degradation. To determine the mechanism by which the *legS2* mutant bacteria replicate in macrophages, we examined the colocalization of the JR32, *dotA*, and *legS2* mutants with lyso- tracker Red, an acidic dye used to track the lysosome. (Fig 4A) shows images obtained by the Confocal Laser Scanning Microscopy (CLSM), and (Fig 4B) shows the score of the percentage of bacteria colocalized within the lyso-tracker positive compartment. Approximately 60% of the translocation-defective *dotA* mutant was delivered to the lyso tracker-labeled compartment within 1 h of infection. In addition, 52% of the JR32 were destined to the lyso tracker labeled compartment. This is due to the fact that WT cells are restrictive to *L*. *pneumophila* replication. The trafficking of the *legS2* mutant was comparable to that of the JR32 with ~57% lyso tracker labeled compartment. Therefore, the *legS2* mutant’s substantial replication in WT macrophages is not attributed to enhanced evasion of LCV-lysosome fusion.

### The increased replication of the *legS2* mutant in BMDMs is associated with reduced cytokine production

**Fig 5:**
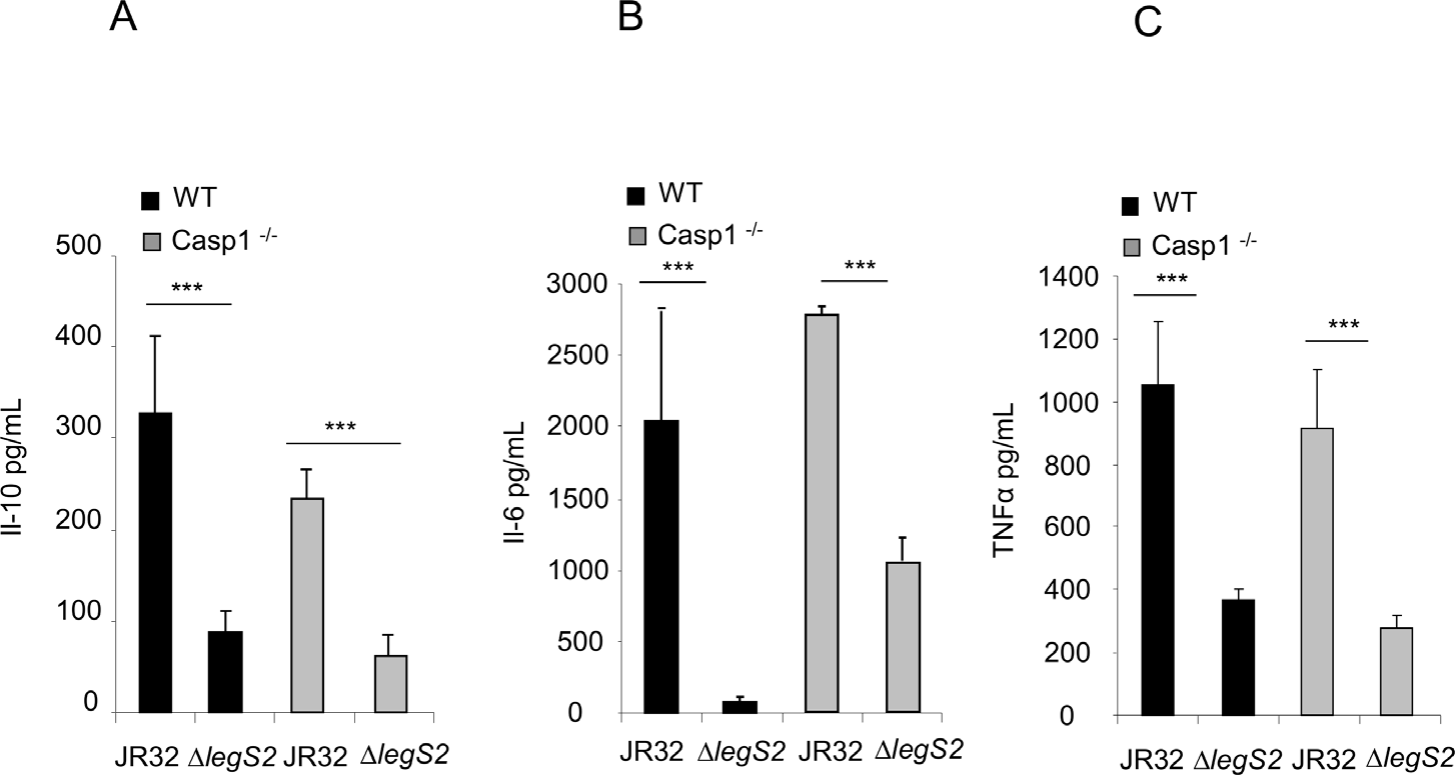
Increased replication of the *legS2* mutant in BMDMs is associated with reduced cytokine production. WT BMDMs or casp-1^-/-^ macrophages were infected with *L*. *pneumophila*, JR32, or the *legS2* mutant. 24 h post infection, the supernatants from infected and un-infected cells were assayed by ELISA. **(**A) IL-10 level, (B) IL-6, (C) TNFα levels. Data represent the mean + SD of n = 3. Asterisks indicate significant differences (***P<0.001), and a two tailed t-test was used to calculate the *P*-value.

The production and secretion of cytokines and chemokines is concomitant with *L*. *pneumophila* intracellular growth [46]. Cytokines are chemical mediators that play fundamental roles in eliciting the necessary immune response for controlling lung infection. We examined the level of several pro-inflammatory and anti-inflammatory cytokines following infection with either the JR32 or the *legS2* mutant by ELISA. After 24 h, the levels of the cytokines IL-10, IL-6, and TNFα were significantly less in macrophages infected with the *legS2* mutant as compared to those infected with JR32. The level of IL-10 in JR32 infected cells was double the amount in *legS2* infected cells, (Fig 5A), the levels of IL-6 and TNFα in JR32 infected cells were triple the amount in *legS2* infected cells, (Fig 5B and 5C). Differences in the cytokine levels were also evident in the Casp1^-/-^ BMDMs 24 h post-infection. Our data suggested that cytokine production is associated with increased replication of the *legS2* mutant in WT BMDMs.

### The *legS2* mutant infected macrophages exhibited reduced cytokine mRNAs accompanied by inhibition of the NF-κB pathway

**Fig 6:**
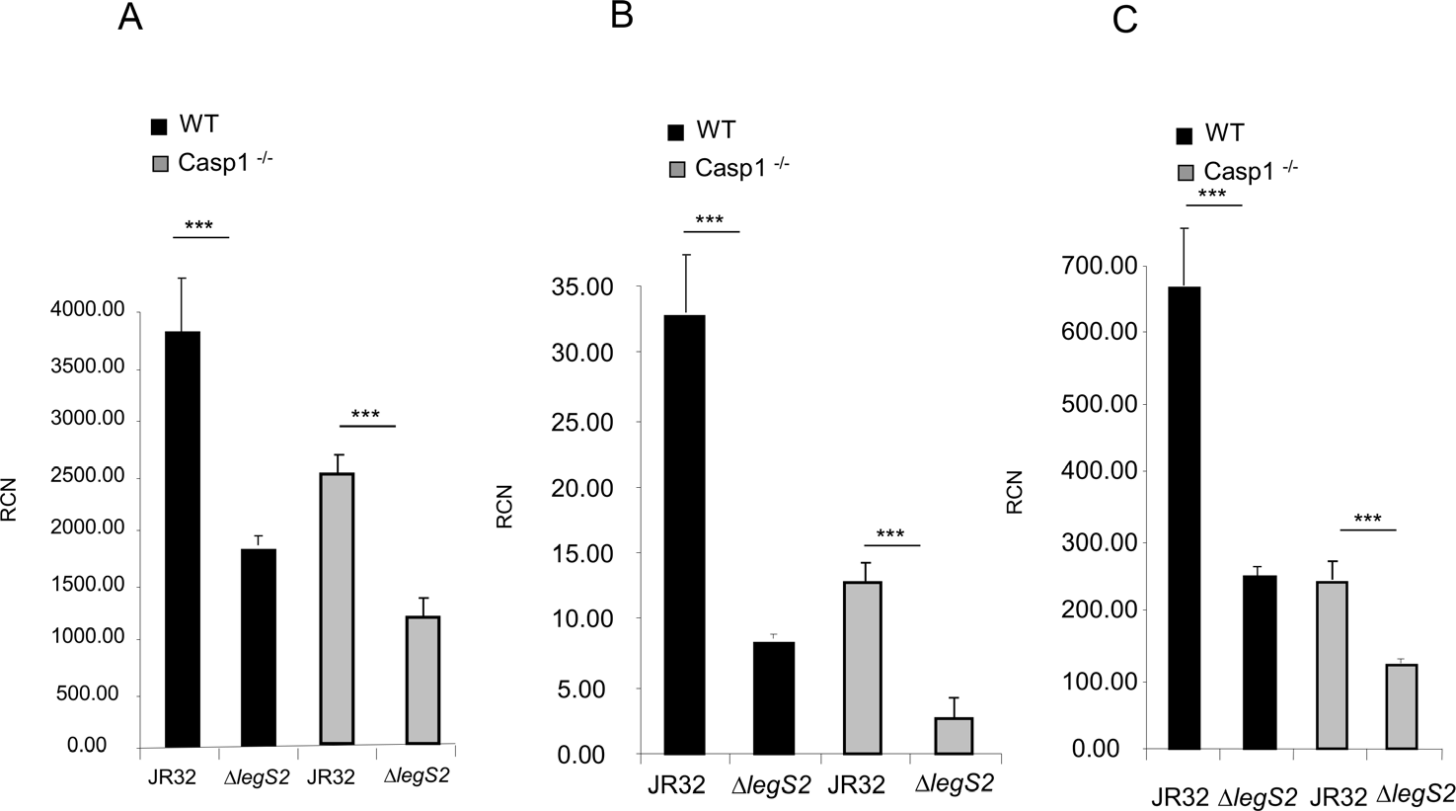
The *legS2* mutant limits the levels of cytokine mRNAs. WT BMDMs or casp-1-/- macrophages were infected with *L*. *pneumophila*, JR32, or the *legS2* mutant. 4 h post infection, total RNA was isolated from JR32 or *legS2* infected macrophages; then, the converted cDNA was used for quantitative PCR. The target gene Ct values were normalized to the Ct values of two housekeeping genes (human GAPDH and CAP-1, according to the cell origin) and expressed as relative copy numbers (RCN). (A) Represents the IL-10 RCNs, (B) Represents the IL-6 RCNs, (C) Represents the TNFα RCNs. Data represent the mean + SD of n = 3. Asterisks indicate significant differences (***P<0.001); a two tailed t-test was used to calculate the *P*-value.

To decipher the mechanism by which *legS2* affects cytokine level, we used quantitative RT-PCR to examine the levels of the cytokine mRNA following infection with either the JR32 or the *legS2* mutant. At 4 h post-infection, the transcription levels of IL-10, IL-6 and TNFα were reduced in the *legS2* infected monolayers as compared to the JR32 infected monolayers (Fig 6A–6C). Our results indicate that the decrease in cytokine level from the *legS2* infected cells was due to lower levels of cytokine mRNA.

**Fig 7:**
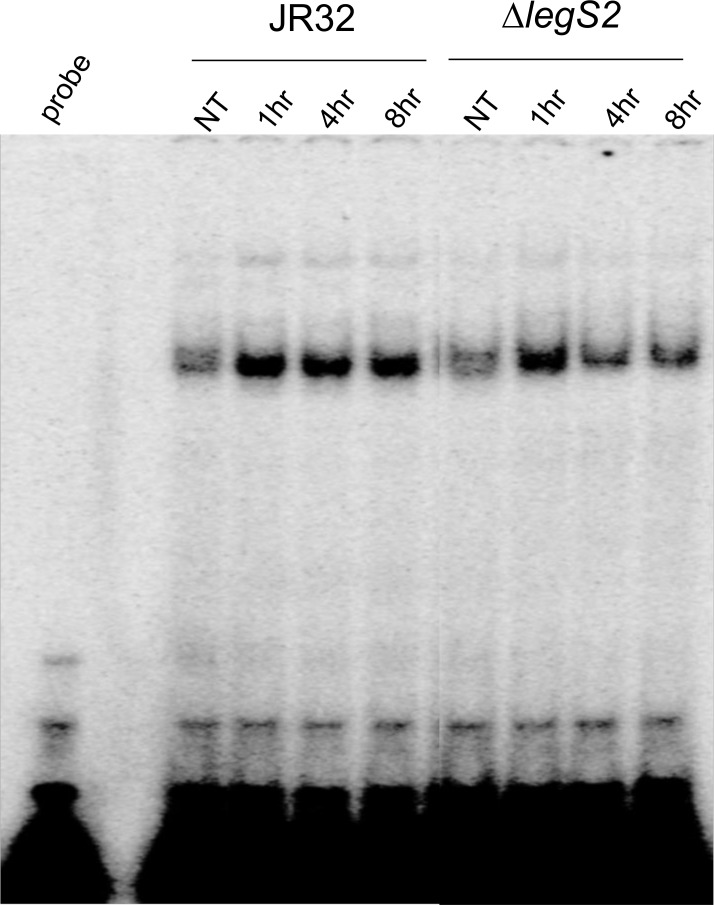
The *legS2* mutant inhibits the NF-κB pathway. Wild-type macrophages were either not treated (NT) or infected with the JR32, or the *legS2* mutant for 1, 4, and 8h. Then, nuclear extracts were processed using electrophoretic mobility shift assay (EMSA) to determine NF-κB activation.

NF-κB is a transcription factor that regulates the mammalian innate immune system by modulating the transcription of pro-inflammatory cytokine and chemokine genes [47]. Indeed, infection with *L*. *pneumophila* activates the host NF-κB pathway [1,48]. Therefore, we examined NF-κB activation by an electrophoretic mobility shift assay (EMSA) (Fig 7). The EMSA data showed that, 4–8 h post-infection, NF-κB was inhibited in the *legS2* mutant and not in the JR32 strain. This suggests that the reduced cytokine production is associated with the inhibition of the NF-κB pathway.

### Infection with the *legS2* mutant is accompanied by change in mitochondrial morphology

**Fig 8:**
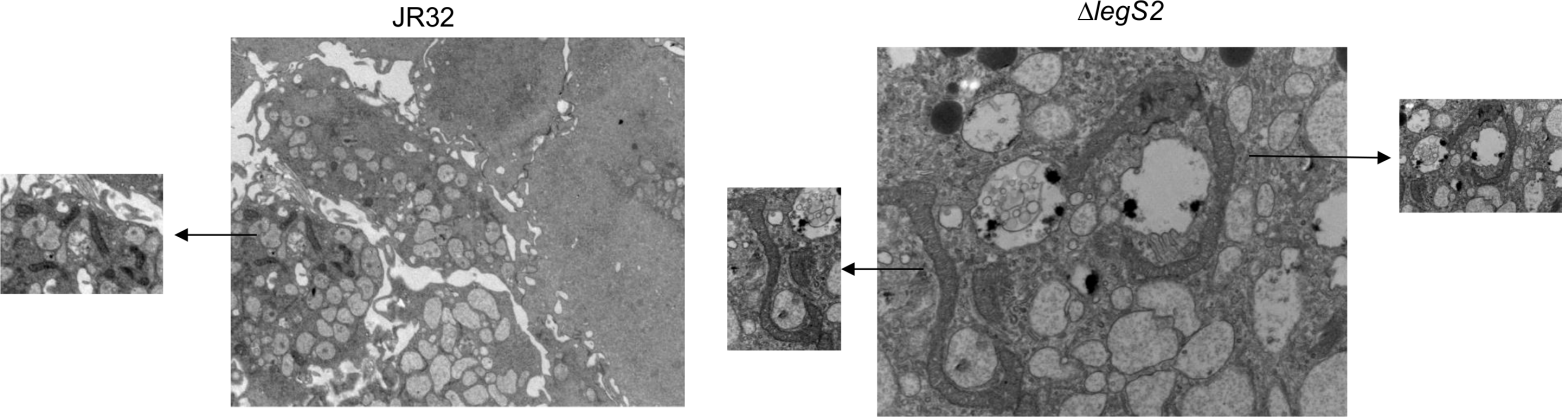
Infection with the *legS2* mutant is accompanied by change in mitochondrial morphology. TEM of BMDMs infected with the JR32 or the *legS2* mutant. Images were taken from 24 h post-infected cells.

The dynamic nature of mitochondrial morphology is shown by its continuously fusing and interconnecting network [49]. The fusion process is critical for the maintenance of mitochondrial function [50]. However, mitochondrial fission is an early event during apoptosis, either occurring before or coinciding with cytochrome c release [49,51,52]. It is upstream of caspase activation and characterized by mitochondrial fragmentation into multiple small units [49,51,52]. We employed transmission electron microscopy to examine cells infected with either the JR32 or the *legS2* mutant at 4 and 24 h post-infection (Fig 8). Our data showed that, 24 h post-infection, BMDMs infected with the *legS2* mutant exhibited elongated mitochondria that seemed to be fused with each other. In BMDMs infected with the JR32 strain, however, we observed fragmented mitochondria, indicating fission. These data suggest that the *legS2* mutant bacteria manipulate mitochondrial fusion and fission, respectively.

## Discussion

In summary, we show that the *legS2* mutant bacteria replicate in WT mice and their derived macrophages. The permissiveness of BMDMs to infections with the *legS2* mutant, is accompanied by the dampening of pro-inflammatory and anti-inflammatory cytokines, inhibiting the NF-κB pathway resulting in changes in mitochondrial morphology.

Studies with the A/J-derived mouse macrophages, which are permissive to *L*. *pneumophila* infection, showed a biphasic activation of NF-κB during infection [1,48,53,54]. The early phase is characterized by strong but transient activation. Even though this phase is beneficial for the host cell, it must be dampened quickly to overcome the toxic effect of inflammatory cytokines [54]. IL-6 and TNFα are major proinflammatory cytokines that are produced in response to NF-κB activation. They regulate inflammatory responses locally and systemically. Moreover, they recruit leukocytes and mediate more cytokine production and increased bactericidal activity [55,56]. The limitation of the above cytokines following infection with the *legS2* mutant is likely responsible for the enhanced *L*. *pneumophila* growth *in vivo*. Indeed, patients with Legionnaires’ disease show increased level of IL-6 and TNFα in their lungs [57–60]. Furthermore, the addition of TNFα renders macrophages less permissive for *L*. *pneumophila* and renders neutrophils more bactericidal [61] [62]. Also, mice with a mutation that abrogates Toll-like receptors (TLRs) exhibit limited cytokines, which in turn results in increased susceptibility to *L*. *pneumophila* lung infection [63–65]. Therefore, the reduction in the levels of IL-10, IL-6 and TNFα could result in the increased replication of the *legS2* mutant.

We also observed that infecting BMDMs with *legS2* mutant results in the reduction of IL-10 level, and other studies have shown that IL-10 is induced *in vivo* upon *L*. *pneumophila* infection [57,59,66–68]. IL-10 is an anti-inflammatory cytokine that represses the expression of Th1 cytokines [69–71]; therefore, it seems that, by dampening the IL-10, the *legS2* mutant reduces *L*. *pneumophila* replication. However, it is quite likely that limiting IL-6 and TNFα outweighs the effect of IL-10. Thus, the overall impact of a *legS2* mutant infection is promoting growth in BMDMs. Even though we focused our study on IL-6, IL-10, TNFα and IL-1β, it is likely that *legS2* affects other cytokines that are produced by macrophages as well.

Based upon our RT-PCR analysis of IL-6, IL-10 and TNFα in macrophages infected with *legS2* mutants, we can conclude that infection with *L*. *pneumophila legS2* dampens the mRNA expression of cytokines in infected cells. The reduction in the above cytokines is likely a consequence of the inhibition of the NF-κB pathway seen by EMSA.

Our TEM data show that infection with *legS2* mutant bacteria results in changes in mitochondrial fusion or fission. We observed mitochondrial morphologies that resemble fused mitochondria in *legS2* infected cells. Mitochondrial fusion has been linked to an increase in the total pool of ATP during starvation [51]. Consistent with our results, Degtyar et. al showed that *legS2* colocalizes to the mitochondria in U937 infected cells [31].

*Listeria monocytogenes* is another pathogen that has been shown to manipulate mitochondrial fusion/fission. The link between mitochondrial dynamics and the efficiency of the *L*. *monocytogenes* infection has been demonstrated. Indeed, the inhibition of the mitochondrial fusion has been shown to decrease the efficiency of *L*. *monocytogenes* infection [72]. Similarly, inducing mitochondrial fusion the *legS2* mutant resulted in a more efficient infection than in the WT. Therefore, modulating mitochondrial fusion/fission by these two pathogens could impact the efficiency of macrophage infection.

We show that *legS2* mutant replicated more than the JR32 strain.

It seems contradictory that *L*. *pneumophila* would maintain *legS2* in the genome, even though it is dispensable for replication in *Acanthamoeba castellanii* [31]. It is possible that retaining redundant genes in the genome of *L*. *pneumophila* is a factor utilized during evolution to prevent chromosomal lesions. Such lesions could affect the ability of the bacteria to multiply intracellularly [30,73]. Moreover, the survival of *L*. *pneumophila* in several protozoan hosts could improve the hosts’ tropism. However, it could prevent host specialization by retaining pathways that are essential for growth in macrophages and other hosts. Therefore, growing *L*. *pneumophila* in laboratories or in murine macrophages, which are not the natural host for this bacteria, could greatly affect the fitness and host range of *L*. *pneumophila* [74]. Furthermore, the restriction of *L*. *pneumophila* growth in murine macrophages by the acquisition of *legS2* could select for bacteria that has increased adaptability to other hosts.
